# Pentraxins and Fc Receptor-Mediated Immune Responses

**DOI:** 10.3389/fimmu.2018.02607

**Published:** 2018-11-13

**Authors:** Jinghua Lu, Carolyn Mold, Terry W. Du Clos, Peter D. Sun

**Affiliations:** ^1^Structural Immunology Section, Laboratory of Immunogenetics, National Institute of Allergy and Infectious Diseases, National Institutes of Health, Rockville, MD, United States; ^2^Department of Molecular Genetics and Microbiology, Albuquerque, NM, United States; ^3^Department of Internal Medicine, University of New Mexico, Albuquerque, NM, United States; ^4^VA Medical Center, Albuquerque, NM, United States

**Keywords:** pentraxin, CRP, SAP, Fc receptor activation by pentraxin, structure and function

## Abstract

C-reactive protein (CRP) is a member of the pentraxin family of proteins. These proteins are highly conserved over the course of evolution being present as far back as 250 million years ago. Mammalian pentraxins are characterized by the presence of five identical non-covalently linked subunits. Each subunit has a structurally conserved site for calcium-dependent ligand binding. The biological activities of the pentraxins established over many years include the ability to mediate opsonization for phagocytosis and complement activation. Pentraxins have an important role in protection from infection from pathogenic bacteria, and regulation of the inflammatory response. It was recognized early on that some of these functions are mediated by activation of the classical complement pathway through C1q. However, experimental evidence suggested that cellular receptors for pentraxins also play a role in phagocytosis. More recent experimental evidence indicates a direct link between pentraxins and Fc receptors. The Fc receptors were first identified as the major receptors for immunoglobulins. The avidity of the interaction between IgG complexes and Fc receptors is greatly enhanced when multivalent ligands interact with the IgG binding sites and activation of signaling pathways requires Fc receptor crosslinking. Human pentraxins bind and activate human and mouse IgG receptors, FcγRI and FcγRII, and the human IgA receptor, FcαRI. The affinities of the interactions between Fc receptors and pentraxins in solution and on cell surfaces are similar to antibody binding to low affinity Fc receptors. Crystallographic and mutagenesis studies have defined the structural features of these interactions and determined the stoichiometry of binding as one-to-one. Pentraxin aggregation or binding to multivalent ligands increases the avidity of binding and results in activation of these receptors for phagocytosis and cytokine synthesis. This review will discuss the structural and functional characteristics of pentraxin Fc receptor interactions and their implications for host defense and inflammation.

## Introduction

**Figure 1:**
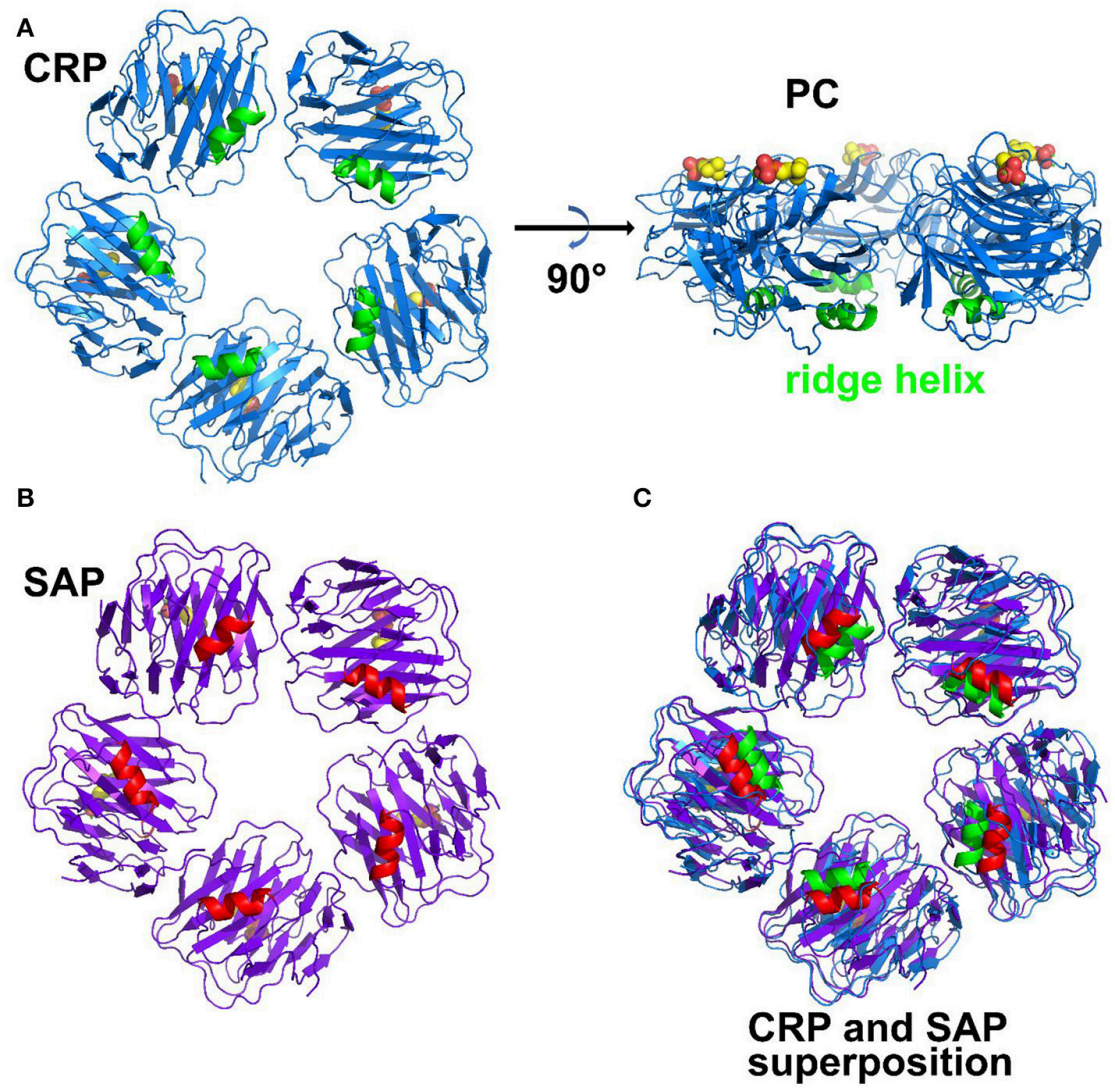
Structure of pentraxins. **(A)** Pentameric structure of CRP. The ridge helix is highlighted in green and the bound phosphocholine molecules are shown in bold sticks. **(B)** Pentameric structure of SAP with ridge helix highlighted in red. **(C)** structural superposition of CRP and SAP (Protein Data Bank ID: 1B09, 1SAC).

Pentraxins are an ancient family of serum proteins that are part of the innate immune system. Pentraxins are defined by a homologous pentraxin (PTX) domain of ~200 amino acids that contains a calcium-dependent ligand-binding site. The two classical pentraxins, C-reactive protein (CRP) and serum amyloid P component (SAP) are composed of non-covalently linked subunits arranged in a planar cyclic pentamer or hexamer (Figure 1) (1). Both CRP and SAP are present in most mammalian species and are represented in evolution as early as the horseshoe crab. A group of proteins designated “long” pentraxins contains the PTX domain along with an additional N-terminal domain (2, 3). This review will focus on the classical or “short” pentraxins, CRP, and SAP, and more specifically the mouse and human proteins and receptors, where the most complete functional studies of CRP and SAP have been done (4, 5).

Pentraxins are pattern-recognition molecules with specificity for damaged cell membranes, nuclear components and microbial antigens (Table 1 comparing CRP and SAP) (6–9). The prototypic ligand for CRP is phosphocholine (PC) (Figure 1). SAP binds phosphoethanolamine (PE) and a number of other ligands, including microbial polysaccharides. CRP was first characterized and named for its binding to the cell wall C-polysaccharide of *Streptococcus pneumoniae*. SAP was initially identified as the precursor for a shared component of amyloid. Each subunit has a single ligand binding site allowing multivalent binding on one face of the pentamer. Multivalent binding by pentraxins initiates complement activation through the classical pathway (10) and promotes recognition and activation of cellular receptors.

**Table 1:** Comparison between CRP and SAP in human and mouse.

	**Human CRP**	**Human SAP**	**Mouse CRP**	**Mouse SAP**
Pentameric structure	Yes	Yes	Yes	Yes
Calcium-dependent ligand binding	Yes	Yes	Yes	Yes
Ligands	PC, C-polysaccharide	PE, LPS	PC, C-polysaccharide	PE, LPS
Nuclear antigen binding	snRNP, histones	DNA, chromatin	Unknown	Unknown
Found in amyloid	No	Yes	No	Yes
Acute phase reactant	Yes	No	No	Yes
Baseline concentration	< 1 μg/ml	30 μg/ml	< 1 μg/ml	10–100 μg/ml varies by strain
FcγR binding	Yes	Yes	Not measured	Not measured
FcαR binding	Yes	Yes	Not measured	Not measured

CRP and SAP are serum proteins, synthesized in the liver, but differ from each other in expression as well as binding specificity. In humans, SAP is expressed constitutively at moderate concentrations (30 μg/ml) whereas CRP is an acute phase protein that increases dramatically in concentration from < 1 μg/ml to several hundred μg/ml during acute inflammation. In the mouse CRP is only found at low concentrations and SAP is an acute phase reactant (11, 12) (Table 1).

**Figure 2:**
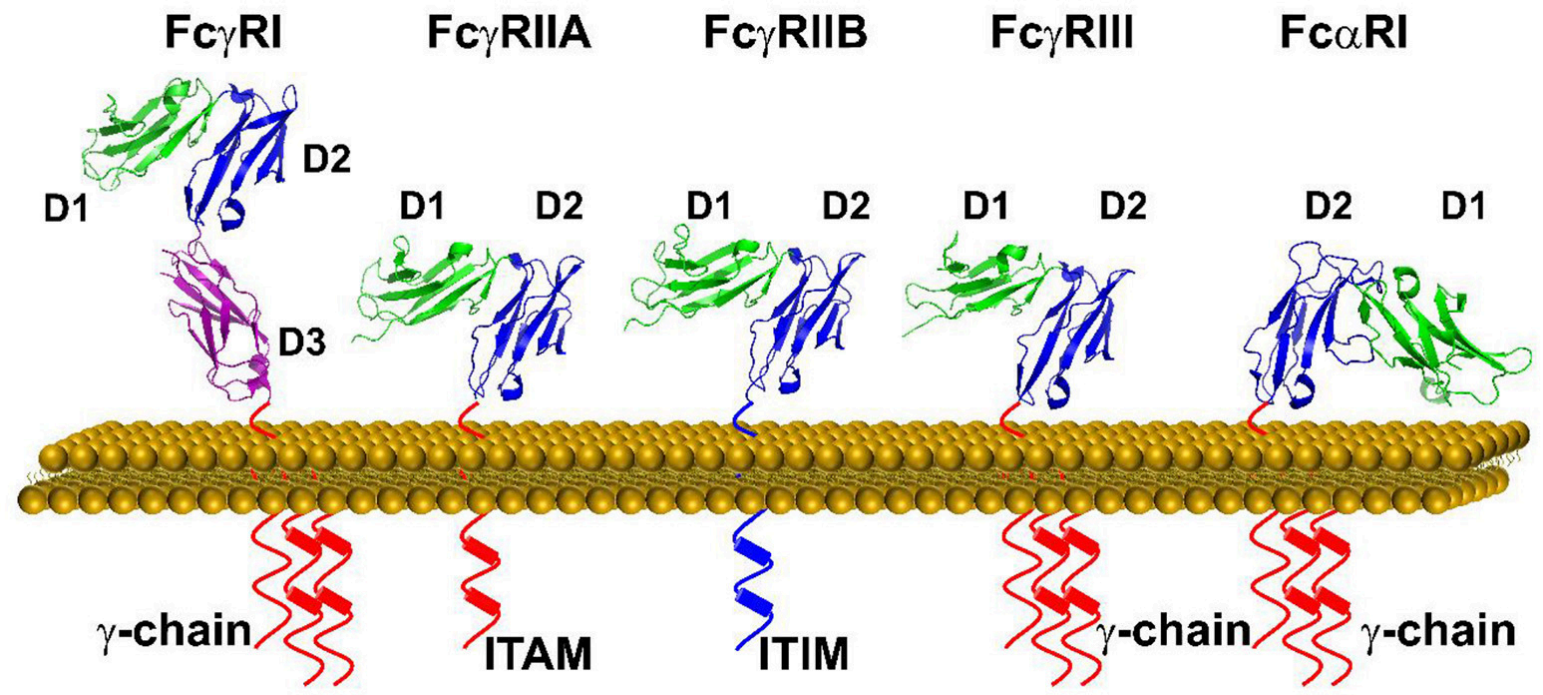
A schematic representation of FcγRs and FcαRI on the cell surface. Each tyrosine residue on the cytoplasmic immuno-tyrosine activating motif (ITAM) or immune-tyrosine inhibitory motif (ITIM) are represented by a cylinder. The orientation of each Ig domain in different Fc receptors is based on the structural superposition of the second Ig domain (D2) (Protein Data Bank ID: 3RJD, 1FCG, 2FCB,3AY4, and 1UCT).

**Figure 3:**
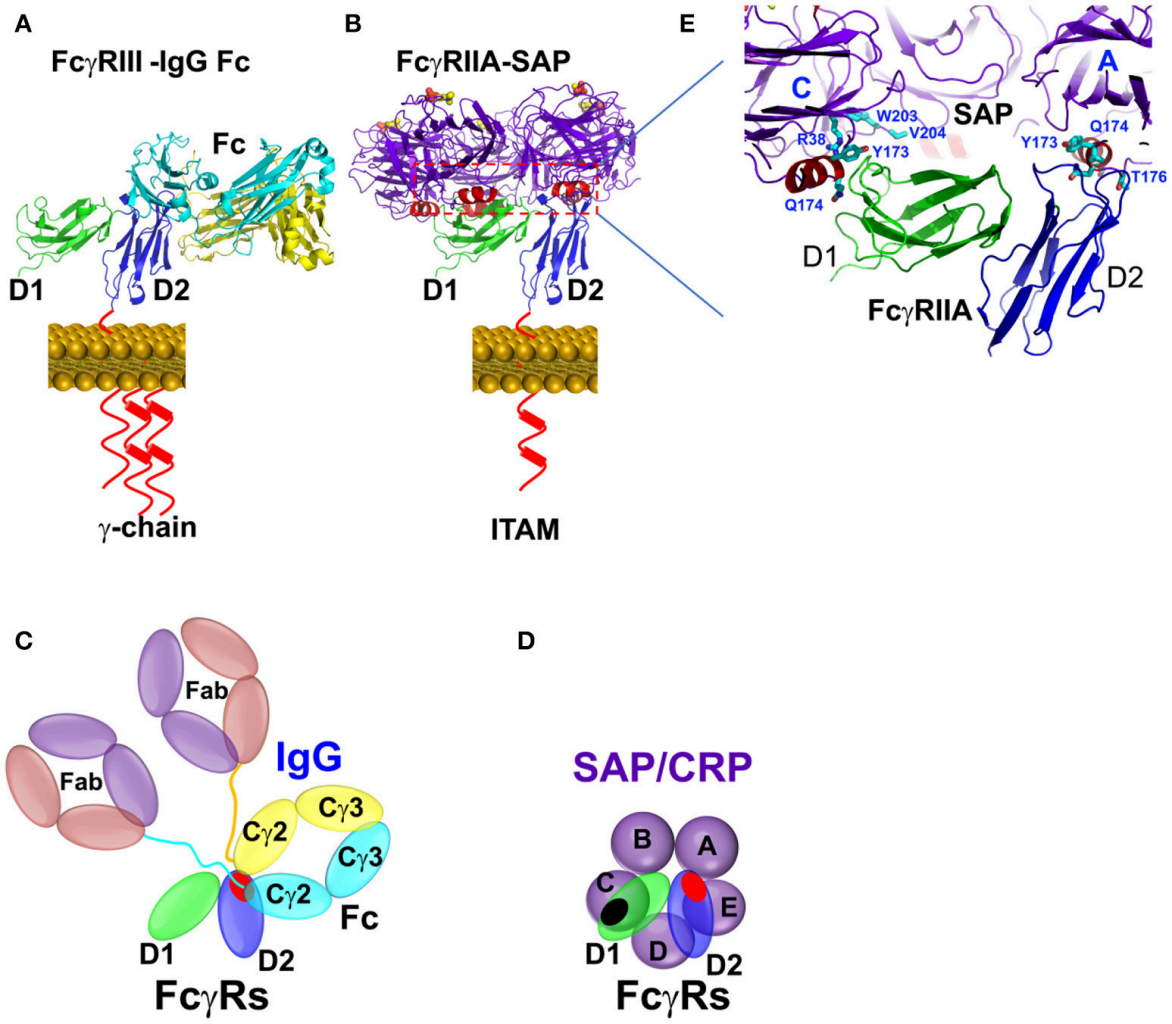
Binding mode between Fcγ receptors and pentraxins or IgG antibody. **(A)** The FcγR-IgG Fc interaction was represented by FcγRIIIA-IgG1 Fc complex structure (PDB ID: 1T83). **(B)** The complex structure between SAP and FcγRIIA(PDB ID: 3D5O). **(C,D)** Schematic representation of the interaction between pentraxin-FcγRIIa, IgG1 Fc-FcγRIIa, or FcγRIII. The interfaces are highlighted in shaded red or black circles. **(E)** Binding sites of FcγRIIA on SAP in crystal structure (PDB ID: 3D5O).

Immunoglobulin Fc receptors (FcRs) expressed primarily on hematopoietic cells provide essential links between antibody and cellular responses (13, 14). FcR are named for their specificity for different isotypes of immunoglobulins. Structurally IgG receptors, FcγR, as well as the IgE receptor, FcεRI, and the IgA receptor, FcαRI, are members of the immunoglobulin superfamily with two or three C2-type immunoglobulin-like extracellular domains (Figure 2). There are multiple human FcγR including the high affinity receptor FcγRI, and several low affinity receptors, FcγRIIa, FcγRIIb, FcγRIIIa, and FcγRIIIb, that differ in cell expression and associated signaling pathways. Despite a high degree of sequence identify in their extracellular domains, FcγR have distinct IgG subtype specificities as well as differences in affinity for IgG. FcγR crosslinking is required for signaling through either activating motifs (immunoreceptor tyrosine-based activation motifs, ITAM) or inhibitory motifs (immunoreceptor tyrosine-based inhibitory motifs, ITIM) found in receptor cytoplasmic domains or associated signaling chains (Figure 2). This restricts cellular responses such as phagocytosis, cytokine synthesis, and cytolysis to IgG in complex with multivalent antigen. Several structures of the extracellular portions of FcR have been published and similar modes of binding to immunoglobulin Fc domains have been defined (15–19). In all cases, one Fc receptor interacts with both heavy chains of Fc asymmetrically in the lower hinge region of IgG between CH1 and CH2 domains (Figure 3). This Fc receptor binding induces a conformational change in the relative orientation of the two antibody heavy chain CH2 domains, such that the two-fold symmetrically positioned CH2 domains observed in the receptor-free antibody structures become asymmetrically positioned to the bound Fc receptor. This obligates immune complex formation as the means for antigen aggregation.

## Identification of FCγ receptors as pentraxin receptors

Opsonization of bacteria and complement activation were the earliest recognized activities of CRP (10, 20). Using C-polysaccharide-coated erythrocytes as targets, CRP was shown to promote phagocytosis both directly and via complement activation (21). CRP-dependent phagocytosis was inhibited by aggregated IgG. Despite these functional data, identification of the cellular receptors for CRP was controversial for many years. These studies were complicated by the expression of multiple FcγR on different hematopoietic cells. A comparison of the presence of different FcγR on cells with CRP receptors suggested that human FcγRIIa was a major CRP receptor. The recognition between pentraxins and FcγR were established using COS cells transfected with FcγRI and FcγRIIa (22), demonstrating FcγRIIa as the primary CRP receptor on human monocytes (23). Additional studies found that SAP also bound to FcγR to promote phagocytosis (24). In subsequent studies both human CRP and SAP bound and activated human and mouse FcγR on monocytes, macrophages and neutrophils (4, 25). Interestingly, CRP binding to FcγRIIa, the predominant receptor on neutrophils and monocytes, was found to be allele-specific (26). FcγRIIa is found in two allelic variants, which differ in a single amino acid (R or H) at position 131. CRP binding is specific for the R variant whereas IgG binds with higher affinity to the H allelic form.

## Structural recognition of pentraxins by FCγ receptors

Using a solution BIAcore-based binding assay, the binding affinities between CRP, SAP, and the long pentraxin PTX3 and FcγR were examined systematically (Table 2). The results showed both a general ~μM binding affinity between pentraxins and FcγR as well as isoform dependent differences (25). Solution studies using isothermal calorimetry established the affinity for the CRP-FcγRIIa interaction as 4 μM with a stoichiometry of 1:1. As previously seen in experiments with peripheral blood cells from FcγRIIa-typed individuals, CRP recognition of FcγRIIa was limited to the R allelic variant.

**Table 2:** Binding affinity between pentraxins and human Fc receptors in solution (25).

	Dissociation constants Kd (μM)				
	**CRP**	**SAP**	**PTX3**	**IgG1**	**IgA**
FcγRI	3.2	0.5	n.d.	0.03	–
FcγRIIa	1.9	1.4	19	0.32	–
FcγRIIb	4.1	1.2	n.d.	0.64	–
FcγRIII	4.1	2.9	1.6	0.38	–
FcαRI	2.8	3.2	n.d.	–	0.12

*n.d. means Not detectable*.

Structural characteristics of the interaction between pentraxins and FcγRs were determined from the crystal structure of the complex between human SAP and FcγRIIa (Figure 3) (25). FcγRIIa docks across the face of SAP opposite the ligand binding face contacting two of the five pentraxin subunits. The diagonal docking structure of FcR on SAP precludes additional receptor from binding to the pentraxin and ensures a 1:1 stoichiometry between the pentraxin and the receptor despite the presence of five identical receptor binding epitopes. There are no significant conformational changes in either SAP or the receptor. The contact area of the two SAP subunits is approximately equal and similar residues are involved, including Tyr 173 and Gln 174 from the ridge helix and residues 200–204 from the C-terminus. Since Fc receptors have a shared structural fold consisting of two tandem Ig-like domains and CRP and SAP share a cyclic pentameric structure, it is likely that the characteristics of the SAP-FcγRIIa co-crystal apply to other pentraxin-FcγR interactions. This mode of binding for CRP to FcγR is consistent with mutations of the putative interface residues Tyr 175 and Leu 176 of CRP impaired FcR binding (27).

The pentraxin binding partially overlap with the IgG binding sites on the receptor. This is consistent with cellular studies showing the inhibition of CRP and SAP function by IgG.

## Activation of FCγR by pentraxins

Pentraxin binding to peripheral blood cells leads to signaling, opsonization and cytokine production. The opsonic activity of CRP was recognized soon after its discovery as a pneumococcal binding protein. CRP increased phagocytosis of C-polysaccharide coated erythrocytes by both complement-dependent and complement-independent mechanisms (21). Aggregated IgG inhibited CRP-dependent phagocytosis. More recently, the direct participation of FcγR in pentraxin-mediated phagocytosis was shown by the co-localization of FcγRIIa with CRP and SAP-opsonized zymosan during phagocytosis by human macrophages. The pentraxin-opsonized zymosan uptake was inhibited by human IgG (25).

Pentraxins also affect leukocyte cytokine production. Many of the ligands for CRP and SAP directly activated toll-like receptors (TLR), and CRP enhanced proinflammatory cytokine production by human peripheral blood mononuclear cells (PBMC) responding to *S. pneumonia* (28). Cytokine responses of PBMC from individuals homozygous for the R-131 allele of FcγRIIa were more affected by CRP than responses of PBMC from individuals homozygous for the H-131 allele. The ability of SAP to induce cytokines (IL-6, IL-8, IL-10) independently of TLR activation was shown using macrophages from mice genetically deficient in Myd88 or RIP2 to prevent TLR and NOD pathway signaling (25).

Additional *in vitro* activities of pentraxins mediated through FcγR are under further investigation. A limiting role for CRP in dendritic cell maturation and T cell activation in a mouse model of experimental autoimmune encephalomyelitis (EAE) was shown to require the inhibitory receptor FcγRIIb (29). Anti-inflammatory and anti-fibrotic activities of SAP on neutrophils and monocyte/macrophage differentiation mediated through FcγR have been reviewed recently (30). The development of SAP as a therapeutic agent for renal and pulmonary fibrosis is discussed further below.

## Binding and activation of FCαR by pentraxins

**Figure 4:**
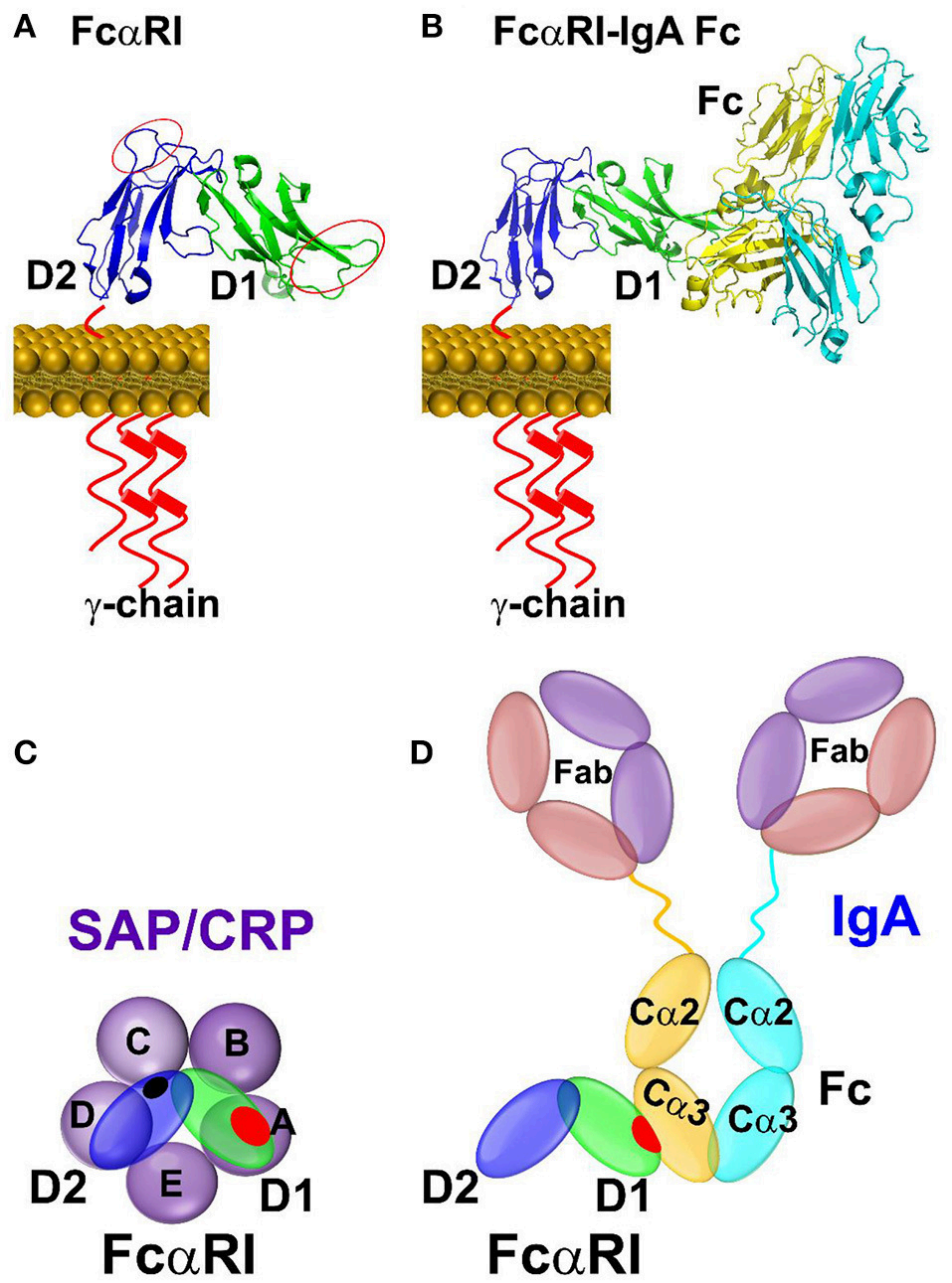
Binding mode between FcαRI and pentraxins or IgA antibody. **(A)** The binding sites identified by site-directed mutagenesis between CRP and FcαRI were indicated by red circles on D1 and D2 domain of FcαRI. **(B)** The complex structure between FcαRI and IgA Fc(PDB ID: 1OW0). **(C)** Schematic representation of the interaction between pentraxin-FcαRI based on site-directed mutagenesis and docking studies. **(D)** Schematic representation of the interaction between IgA Fc-FcαRI in crystal structure (PDB ID: 1OW0). The interfaces are highlighted in shaded red or black circles.

While FcγRs mediate cellular function of IgG, FcαRI, and FcεRI are high affinity receptors for IgA and IgE, respectively. Both FcαRI and FcεRI consist of two tandem C2-type Ig domains that structurally resemble members of FcγRs (Figure 2). The conserved structures of the pentraxins and the shared structural folds of FcR raise the possibility of a broad recognition between pentraxins and FcR. Indeed, FcαRI but not FcεRI showed binding to CRP and SAP in solution (Table 2) (31). Although the *in vivo* relevance remains to be established, much of the functional evidence for pentraxin interaction with FcαRI were shown using FcαRI transfected RBL (Rat Basophilic Leukemia) cells. CRP not only bound FcαRI on transfected cells, crosslinking by CRP induced ERK (extracellular signal-regulated kinase) phosphorylation, degranulation, and cytokine production in transfected RBL cells. In addition, CRP induced surface expression of FcαRI on neutrophils, resulting in phagocytosis and TNF-α production (31). Interestingly, FcαRI is structurally more similar to members of inhibitory NK receptor (KIRs) rather than FcγRs. In particular, the juxtaposition of the two Ig domains on FcαRI is opposite to that of FcγRs (Figure 2). In addition, the IgA binding by FcαRI is also distinct from that of IgG binding by FcγRs. Unlike FcγRs which bind to the lower hinge region of IgG, FcαRI recognizes the membrane proximal region of IgA CH3 domain (Figure 4) (32). The IgA binding epitope on FcαRI involves the receptor N-terminal D1 domain rather than the IgG recognition by the D2 domain of FcγRs. As a result of the structural difference, CRP binding site on FcαRI appears distinct to that of CRP binding site on FcγRs. Subsequent alanine mutations on the receptor have indicated a number of FcαRI residues important for the pentraxin binding. They include Y35, R48, E49, and R82 on the receptor D1 domain, that partially overlaps with the receptor IgA binding site (Figure 4) (33).

## Pentraxin activation of FCγR *in vivo*

*In vivo* functions of pentraxins have primarily been studied in mouse models using human injected or transgenic human CRP. Mice genetically deficient in individual FcγR or complement components have been used to delineate the role of FcγR in these functional settings. Human CRP and human SAP were shown to mediate phagocytosis through mouse FcγRs (34). In these studies CRP opsonization was mediated by mouse FcγRI. SAP opsonization was mediated by mouse FcγRI and FcγRIII. CRP effects in the mouse may also be mediated by the inhibitory receptor FcγRIIb as described below. Mouse pentraxin binding to mouse FcγRs has not been studied due to the very low levels of expression of CRP in the mouse.

The protective role of CRP in pneumococcal Infection was first described in 1989 (35). Mice injected with human CRP were protected from lethal infection with type III or type IV *S. pneumoniae*. These findings have been reproduced using mice transgenic for human CRP. More recently, the relative contributions of complement and FcγR were investigated in CRP-mediated protection from pneumococcal infection (36). CRP was protective in mice genetically deficient in individual or multiple FcγR. However, CRP did not protect C3 or C4-deficient mice from *S. pneumoniae*.

In contrast to the results in pneumococcal infection, CRP protection in a mouse model of endotoxin shock was completely dependent on FcγR (37). It had previously been shown by others that mice transgenic for rabbit CRP or injected with human CRP, but not SAP were resistant to high dose endotoxin lethality (38). In our studies CRP protected wild type mice, but not mice genetically deficient in the FcR γ-chain. Injection of CRP increased serum levels of the anti-inflammatory cytokine IL-10 in wild type, but not γ-chain deficient mice. Mice deficient in FcR γ-chain lack expression of all activating FcγRs, including FcγRI, FcγRIII, and FcγRIV. An additional role for the regulatory FcγRIIb was found in these studies. Mice deficient in FcγRIIb had similar sensitivity to endotoxin shock as wild type mice. However, CRP treatment of FcγRIIb-deficient mice increased their sensitivity to endotoxin shock that was associated with greatly increased serum levels of the pro-inflammatory cytokines TNF-α and IL-12. These results suggest a complex interaction between CRP and FcγR in the regulation of the inflammatory response generated through TLR in response to endotoxin.

A possible clinical correlate of these experiments was found in a study of patients who were hospitalized following severe trauma (39). Patients expressing the CRP-binding allele of FcγRIIa (R-131) were at decreased risk of sepsis and maintained greater MHC class II expression on monocytes in the period following traumatic injury. Decreased class II expression is associated with poor monocyte activation and increased susceptibility to infection in patients following traumatic injury.

Mouse models of immune thrombocytopenic purpura (ITP) have been used extensively to study IgG-FcγR interactions in immune complex and autoimmune disease. Injection of anti-platelet antibodies (rat monoclonal anti-mouse CD41) induces platelet clearance over a period of 24 h with recovery by 48 h. Injection of either human CRP or human IgG (IVIG) prevented platelet depletion in this model and protection was also seen following transfer of CRP-treated spleen cells (40). The transfer ITP model was used to identify the cells and receptors required for CRP effects on immune complex disease. The results showed that CRP treated splenic or bone marrow-derived macrophages transferred suppression and that FcγRI and *syk* activation were required in the donor cells. The protective effect of CRP and CRP-treated macrophages required FcγRIIb in the recipient mice similar to IVIG-mediated suppression of ITP (41).

CRP injection was also protective in immune complex mediated-nephritis (42). Nephrotoxic nephritis (NTN) was induced by immunizing mice with rabbit IgG followed by injection of rabbit antibody to mouse glomerular basement membrane. CRP was protective in this model by an FcγRI, macrophage, and IL-10 dependent pathway, similar to what was found in the endotoxin shock model.

Together these studies demonstrate that CRP can activate macrophages through FcγR to regulate inflammatory responses. In contrast CRP protection against *S. pneumoniae* infection is primarily mediated by complement activation.

Effects of either injected or transgenic CRP have also been seen in more complex autoimmune mouse models (43, 44, 45). Injected CRP prevented and treated renal disease in two mouse models of SLE (NZBxNZW F1, and MRL/lpr) similar to its effects in NTN. NZBxNZW F1 mice expressing CRP from a transgene showed delayed development of disease. In these cases, the mechanisms of protection are likely to be complex and have not been fully determined. Overproduction of type I interferon by plasmacytoid dendritic cells in response to immune complexes containing autoantibodies and nuclear antigens is an important contributor to SLE. We recently showed that CRP inhibits interferon production by purified human pDC responding to immune complexes containing lupus autoantibodies and nuclear antigens (46). pDC express FcγRIIa which mediates uptake of immune complexes that stimulate the type I IFN response through intracellular TLR (47).

Experimental autoimmune encephalomyelitis (EAE) is a model for multiple sclerosis and has been studied extensively as a T cell-mediated autoimmune disease. CRP expressed from a transgene is protective in mouse EAE by shifting the phenotype of autoimmune T cells from T_H_1 to T_H_2 (48). More recently, CRP was found to inhibit dendritic cell maturation thereby decreasing antigen-driven T cell activation. The effect of CRP on dendritic cells and its effect on EAE *in vivo* required the inhibitory receptor, FcγRIIb (29).

SAP also regulates the inflammatory response by binding to FcγR. SAP promotes phagocytosis and enhances cytokine production by monocytes and macrophages through activating FcγR as described above. In addition, SAP was identified as a serum factor responsible for inhibiting the differentiation PBMC into fibrocytes, and this effect was inhibited by aggregated IgG (49). *In vivo* SAP attenuated fibrosis in both renal and pulmonary models by regulating macrophage polarization (30, 50). Recombinant human SAP is currently in phase II clinical trials for the treatment of myelofibrosis and idiopathic pulmonary fibrosis (51).

## Conclusion

Pentraxins are serum acute phase proteins conserved throughout the animal kingdom. While they are known to activate complement to provide innate immunity against infections, recent work established their role in activating cellular immune functions. Members of the pentraxin family can crosslink and activate a subgroup of Fc receptors upon opsonization, similar to Fc receptor activation by immune-complexes. Because of their ability to activate both soluble and cellular immune responses and their inflammatory nature, pentraxins interface innate, and adaptive immunity. These features are essentially shared with antibodies. However, while antibodies are highly restricted to individual antigenic epitopes, pentraxins are pattern recognition receptors with broad specificity for microbial glycans. They are therefore not redundant but complementary to each other in providing host immune surveillance.

## Author contributions

All authors listed have made a substantial, direct and intellectual contribution to the work, and approved it for publication.

### Conflict of interest statement

The authors declare that the research was conducted in the absence of any commercial or financial relationships that could be construed as a potential conflict of interest.
